# Quantifying Depuration of Methylmercury from Fish Consumption by Travelers

**DOI:** 10.1021/envhealth.5c00389

**Published:** 2025-11-04

**Authors:** Ryan F. Lepak, Jean Hervé Mve Beh, Clotaire Moukegni-Sika, Jean Noël Bibang Binguema, Sarah E. Janssen, Jacob M. Ogorek, Michael T. Tate, Peter B. McIntyre

**Affiliations:** † Center for Computational Toxicology and Exposure, Great Lakes Toxicology and Ecology Division, U.S. Environmental Protection Agency Office of Research and Development, 6201 Congdon Blvd, Duluth, Minnesota 55804, United States; ‡ Laboratoire d’Hydrobiologie et d’Ichtyologie, Centre National de la Recherche Scientifique et Technologique (CENAREST), 206860 Institut de Recherches Agronomiques et Forestières, Libreville BP 2246, Gabon; § Ministères des Eaux et Forêts, Agence Nationale des Parcs Nationaux, Libreville 3641, Gabon; ∥ CENAREST, Institut de Recherches Agronomiques et Forestières (IRAF), Libreville BP 2246, Gabon; ⊥ Upper Midwest Water Science Center, M3 Research Laboratory, U.S. Geological Survey, 1 Gifford Pinchot Drive, Madison, Wisconsin 53726, United States; # Department of Natural Resources and the Environment, 5922 Cornell University, 226 Mann Drive, Ithaca, New York 14853, United States

**Keywords:** Minamata, human biomarker, mercury isotopes, hair, fish

## Abstract

During a two-week field sampling expedition in Gabon, two American scientists consumed fish daily from the Ogooué River watershed. We sampled their scalp and facial hair periodically to evaluate hair as a biomarker to track shifts in methylmercury (MeHg) exposure from diet. Each individual differed in the onset and extent of MeHg accumulation but showed similar depuration rates. Pretrip baseline Hg isotope values between participants were distinct from Gabonese fishes allowing us to detect shifts in MeHg sources in the hair of both individuals. δ^202^Hg values tracked the mass-dependent fractionation of MeHg depuration stemming from *in vivo* metabolism, leading to δ^202^Hg increases of 0.014 ± 0.001 per mille and total Hg losses of 8.3 ± 1.1 ng g^–1^ daily. While limited in scope due to minimal participants, our findings reveal a complex interaction between prior MeHg burdens, contemporary MeHg intakes, and sources of consumed fishes (locally caught versus market-sourced) in determining the dynamics of MeHg concentrations and δ^202^Hg in human hair. We also suggest that the offset in δ^202^Hg values used in literature between fish and human hair (1.75 ± 0.25‰) may overlook a time domain that increases starting fish-hair δ^202^Hg offsets (0.94‰), through time.

## Graphical abstract

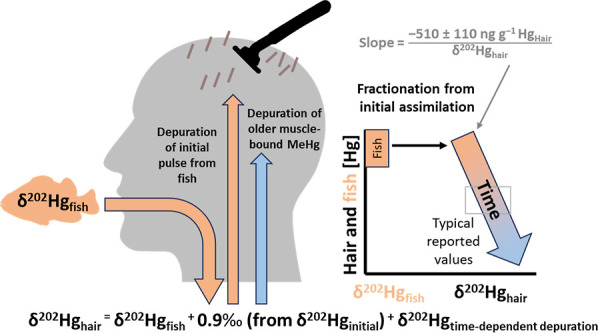

## Introduction

Wild-caught fish are an important source of protein, micronutrients, and essential fatty acids for human populations worldwide, and the accessibility of inland and coastal marine fisheries often makes them a critical source of subsistence for low-income households.^1–4^ Wild fish also can incorporate harmful contaminants that can become biomagnified through the food web.^5,6^ Mercury (Hg) is the most ubiquitous contaminant in fisheries worldwide due to its presence in coal, use in low-technology mining, and capacity for long-distance transport in the atmosphere. In its elemental and divalent forms, Hg has low toxicity and retention in animals, but when microbially converted into methylmercury (MeHg), it becomes both neurotoxic and bioaccumulative.^7^ Aquatic ecosystems receive Hg by both indirect and direct pathways, thereby contaminating fishes in proportion to the ecosystem supply rate, the net methylation rate, and the biomagnification factor, all of which vary widely due to many variables.^5,6,8^

The Minamata Convention on Mercury recommends human biomonitoring using hair, blood and/or urine depending on the source, duration of exposure and the Hg species of interest.^9^ Blood and hair are both commonly used to assess MeHg exposure. Hg in hair reflects exposure over a longer time than blood because it remains bound as the hair continues to grow, essentially creating a time series within each hair. Thus, dietary records can be paired with hair samples to interpret sources of MeHg exposure, since hair Hg is often dominated by MeHg.^9^ Distinguishing among Hg species is important when analyzing hair, to avoid contamination by dust or sorbed inorganic Hg.^9^

Mercury stable isotope ratios have enabled scientists to infer sources and cycling of Hg in the absence of detailed data on pools and fluxes. This Hg science has matured enough that the isotope ratios of many major sources have been characterized,^10^ enabling inferences about contamination pathways based on snapshots of Hg isotopes in field samples even from poorly studied systems. The validity and precision of these inferences rests on the applicability of kinetic and equilibrium reactions measured in benchtop studies and then validated by empirical work.^11–13^ Three classes of fractionation are common for Hg: mass dependent fractionation (MDF; represented by δ^202^Hg), odd-mass independent fractionation (odd-MIF; represented by Δ^199^Hg) and even-mass independent fractionation (even-MIF; represented by Δ^200^Hg).^14^ Interpreting Hg isotope values from hair samples relies upon three additional assumuptions. First, little or no odd- or even-MIF occurs during the trophic transfers in food webs.^15^ Second, comparatively more MDF occurs during trophic transfers in food webs, but the extent of fractionation is minimal compared to natural variation among Hg sources.^13,15^ Third the extent of MDF associated with transfer of MeHg from fish to human hair is constrained (δ^202^Hg is 1.5‰ to 2.0‰ higher in hair).^12,16,17^ But, there is uncertainty to the degree of mercury isotopic fractionation accompanying MeHg assimilation and depuration,^18^ which complicates the interpretation of results from hair samples. This knowledge gap is a key barrier to relying on Hg isotopes from human hair for biomonitoring under the Minamata Convention.

To gain a clearer sense of how fish MeHg is transferred to humans, bioaccumulated and the depuration of Hg in human hair, we collected a time series of hair samples from two individuals who were exposed to a mercury-rich diet of fish during a 14-day field expedition in Gabon. While we recognize two individuals is insufficient to create generalizable information, this unique opportunity allowed us to document, for the first time, natural human exposure to dietary Hg in an environmental setting. Prior to that period, one study subject was a frequent consumer of high-MeHg Lake Superior lake trout^19,20^ while the other occasionally (∼2 times monthly) consumed low-MeHg wild-caught Alaskan salmon.^21^ Both subjects abstained from fish for one month prior to departure, so we expected to observe rapid shifts in the hair Hg concentration and isotope values during the field expedition. Furthermore, we expected that MDF during depuration of MeHg assimilated in Gabon would drive isotope values after returning to the USA. We hypothesized that Δ^199^Hg and Δ^200^Hg would shift toward Gabonese baselines but then remain stable afterward, but δ^202^Hg would shift toward the Gabonese fish baseline during travel and then rise continuously after the trip. We also predicted Hg isotope values in the hair of the more piscivorous subject would be less sensitive to new Hg inputs than those of the infrequent fish consumer, because baseline concentrations would be much higher. Lastly, we hypothesized that MDF of depuration would be similar between the individuals despite their dissimilar Hg baselines.^18^ By taking advantage of a major shift in mercury content and isotope signature of human diets, this study was intended to elucidate the basis for the observed variability in the δ^202^Hg offset between fish and hair in prior studies, which is a key uncertainty in interpreting results of hair sampling under the Minamata Convention.

## Experimental Section

### Field Expedition and Individuals

Both subjects arrived in Libreville on 1 January 2019 to collaborate with the Agence Nationale des Parcs Nationaux in Gabon on a country-wide assessment of Hg in fish. During the two-week field campaign, both individuals traveled together and ate domestic fish. Portion size and serving frequency (∼300 g fresh weight, at least every other day) were comparable, but not precisely quantified. The fish were from the Ogooué River system and are expected to be representative of prevailing Hg concentrations throughout Gabon. The Hg results from a wide variety of commonly eaten fish species in Gabon are summarized in Table S1 and averaged in Figure 1A but will not be discussed further herein. The U.S. Environmental Protection Agency Human Subjects Research Review Official determined that this research is not human subjects research. This determination is based on the fact that the study does not meet the definition of research as the intention was not to develop or contribute to generalizable knowledge.

**Figure 1:**
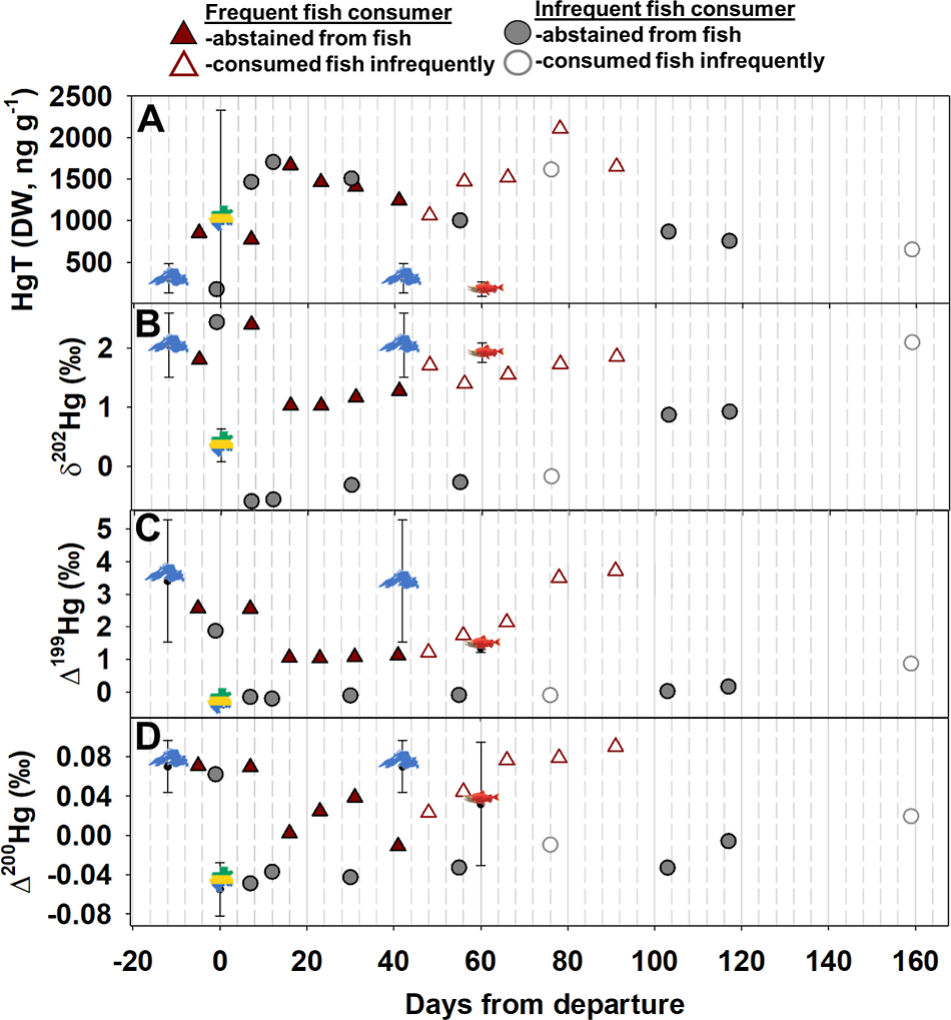
(A) (top) Total Hg (HgT) concentrations (ng g^–1^, dry weight [DW]) in hair and fish. Symbols of Lake Superior,^19,20^ Gabon and an Alaskan sockeye salmon (*Oncorhynchus nerka*)^21^ represent fish HgT data from those respective basins. Both participants consumed Gabonese fish (days 1–14), but the frequent fish consumer also consumed Lake Superior fish whereas the infrequent fish consumer consumed Alaskan salmon, very infrequently. The errors represent 1 standard deviation of that data. Filled triangles and circles represent study-period abstinence from fish consumption for the frequent and infrequent fish consumers, respectively. Open triangles and circles represent a return to consuming fish in the USA following the expedition; the infrequent consumer ate fish only once in 120 days, whereas the frequent consumer often ate fish starting 30 days after their return. (B) δ^202^Hg values in hair and fish. 1.75‰ was added to the fish data to account for the typical fractionation associated with MeHg accumulation and subsequent depuration in hair.^17^ (C) Δ^199^Hg values in hair and fish. (D) (bottom) Δ^200^Hg values in hair and fish.

The two subjects of this study were middle-aged males self-reporting as white, non-Hispanic, but had contrasting dietary MeHg intake histories. One was a frequent consumer of fishes (scalp hair: Hg_ini_ *x̅* = 813 ± 56 ng g^–1^ DW; Table S2), while the other was an infrequent consumer of fishes (facial hair: Hg_ini_ *x̅* = 120 ± 13 ng g^–1^ DW). To sample hair, the frequent fish consumer shaved his scalp whereas the infrequent fish consumer shaved the outer length of facial hair, both at 10-to-14 day intervals. This approach ensured samples represented comparable timespans per sample. Hair was placed in a new polypropylene bag, stored frozen, and lyophilized. We also tested the difference in scalp versus beard hair for the infrequent fish consumer and the isotope values were indistinguishable, but scalp hair showed higher Hg concentration (Hg_ini_ *x̅* = 173 ± 8 ng g^–1^ DW, about 50 ng g^–1^ higher). However, we are presently unable to precisely evaluate whether depuration characteristics are identical between hair types for HgT. For 30 days following the field campaign, neither individual consumed fish. Following 30 days, the frequent fish consumer transitioned back to their normal diet whereas the infrequent fish consumer continued to abstain until day 160, except for one sockeye salmon meal on day 80.

### Analytical Measurements

Mercury analyses were conducted at the U.S. Geological Survey Mercury Research Laboratory. Due to mass constraints; we elected to solely measure HgT concentrations in hair and forego speciated Hg analyses. In doing so, we assume the hair is predominantly MeHg, a reasonable assumption given the participants’ life histories are known and their Hg exposure was dominated by fish consumption. The certified reference material National Institute for Environmental Studies (NIES-13, Japan) was measured three times and averaged 97 ± 2% recovery. For HgT stable isotope analysis, approximately 0.1–0.5 g of hair was digested in 1–5 mL of concentrated nitric acid (95 °C) overnight, oxidized with 10% (v/v) bromine monochloride, and then heated again overnight. The resulting extracts were then diluted to a 20% acid concentration, measured for HgT by cold-vapor atomic fluorescence spectrometry to ensure complete recovery, and then analyzed for HgT stable isotopes.^19,22^ NIES-13 was used as the isotopic Hg reference material and UM Almadén (National Institute of Standards and Technology, NIST RM 8610) was used as a secondary reference standard. δ^202^Hg, Δ^199^Hg and Δ^200^Hg were calculated according to previously published methods and results are in the SI (Table S2).^14^ The analytical error is determined by the maximum deviation in NIES-13 triplicate measurements (±0.12‰ for δ^202^Hg, ±0.04‰ for Δ^199^Hg, and ±0.03‰ for Δ^200^Hg [2SD]—see Table S1).

## Results and Discussion

### Gain and Depuration of MeHg in Hair

When account for MeHg concentration data in Gabonese fishes (Table S1) and the approximate daily portion size, we estimate that both subjects exceeded by 2 to 4-fold the recommended reference dose of MeHg (0.1 μg MeHg kg^–1^ body weight day^–1^).^23^ MeHg is absorbed in the digestive tract and reaches hair and tissues through the circulatory system via thiol-containing biomolecules that aid in biotransport and are concentrated in cystine-rich hair follicles that become a route of depuration (alongside hepatic and renal systems).^18,24^ Depuration in hair is kinetically controlled, but the underlying mechanisms are incompletely understood. The ratio of hair to blood concentrations appears to serve as an indicator of depuration rates; MeHg depuration from hair becomes less efficient as exposure rises.^18^

The incorporation of Gabonese Hg followed a different temporal trajectory in the infrequent (173 ng g^–1^ to 1466 ng g^–1^ in 7 days; facial hair) versus frequent (853 ng g^–1^ to 1668 ng g^–1^ in 16 days; scalp hair) fish consumer. We observed a more rapid pace of Hg assimilation into hair than previously suggested (∼3 weeks; Figure 1A).^18^ We expected to find disparate peak MeHg concentrations arising from differences in pre-Gabon MeHg exposure of the two subjects, but kinetic control of blood-hair exchange^18^ apparently overwhelmed the initial differences. Both individuals, of similar weights and thus likely ingestion and exposure rates, reached similar maximum hair concentrations after 2 weeks. This convergence leads us to speculate that the pulse of high MeHg ingestion rates during the two-week expedition led to saturation of the MeHg binding receptors.^25^

Maximum HgT concentrations and the cessation of new exposure occurred at the same time allowing us to quantify MeHg depuration. Hg isotope values in fish from Gabon were sufficiently differentiated from those communly consumed by the participants in the US (Figure 2A). Subjects showed similar depuration rates (Figure 2B–D) both in isotopes and in HgT. We estimated loss of MeHg using two approaches; using the slope of the declining concentrations through time (Figure 2C) and calculating half-life from fitting an exponential decay function (eqs S1 and S2). The exponential decay models suggest significantly shorter (*p* = 0.031) half-life for the frequent fish consumer (56 ± 6 [SD] days) than for the infrequent fish consumer (89 ± 18 days). However, the exponential decay models fit poorly (*R* ^2^ < 0.5). So, we calculated half-life based upon linear projection to be 88 ± 12 days (Figure 2C). This estimate is only marginally shorter than previously reports (102 ± 31 days).^18^ The stronger linear depuration rates may reflect the short time period evaluated for each participant. The earlier onset of declining hair Hg in the infrequent fish consumer also suggests more efficient initialization of the depuration processes when starting from a low-MeHg baseline, though we cannot rule out the possibility that facial hair is simply more responsive than scalp hair. Nevertheless, depuration rates were comparable between the participants based on the strong fit of the linear model.

**Figure 2:**
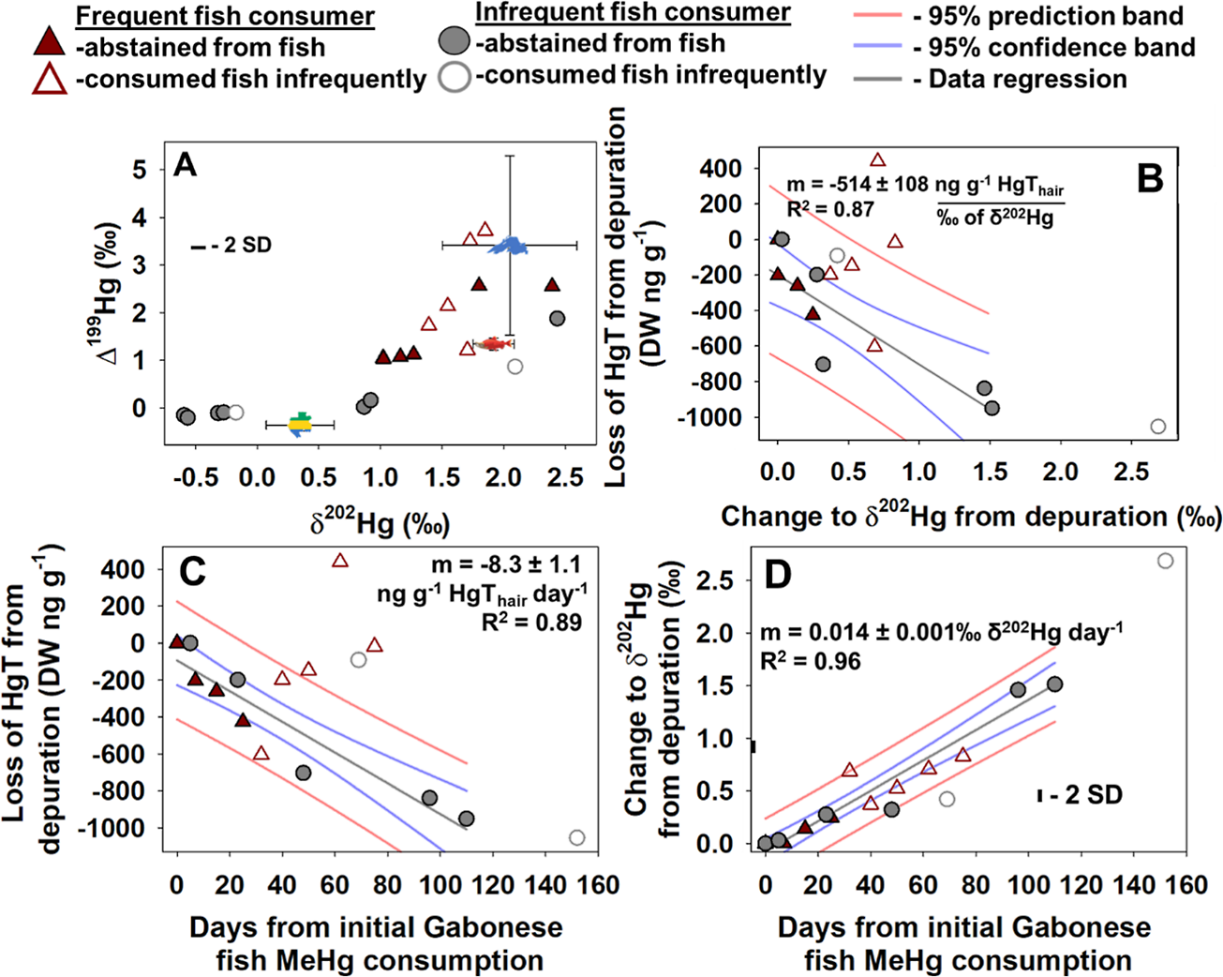
(A) δ^202^Hg values and Δ^199^Hg values (‰) in hair and fish. Symbols of Lake Superior,^19,20^ Gabon and an Alaskan sockeye salmon (*Oncorhynchus nerka*)^21^ represent fish δ^202^Hg values and Δ^199^Hg values from those respective basins. The errors on data symbols represent 1 standard deviation of that data but the standalone error bars represent the 2-standard deviation of certified reference materials. Filled triangles and circles represent study-period abstinence from fish consumption for the frequent and infrequent fish consumers, respectively. Empty triangles and circles then represent time periods where fish was consumed infrequently and for the infrequent fish consumer, only once. This formatting is conserved throughout. (B) Comparing changes in hair δ^202^Hg values (‰) and Hg loss from Hg depuration. The linear relationship is calculated from filled symbols. (C) The change in HgT concentrations though time where the linear calculation only includes the study-period abstinence data for both individuals. (D) The change in δ^202^Hg values though time where the linear calculation only includes the study-period abstinence data for both individuals.

Taken together, our findings on the rapid rise and subsequent slow depuration dynamics of MeHg in human hair suggest that assimilation efficiency is a dynamic consequence of many factors, including initial burden, ingestion rate, and rate of exposure. For contaminated fish, we conclude that uptake of dietary MeHg is inversely related to initial body burdens, either due to disparities in assimilation or depuration rates.^26^ Our two subjects achieved comparable maximum hair MeHg despite large differences in initial concentrations. Understanding such differences in the effective bioavailability of MeHg across a wide range of body burdens is critical for developing appropriate fish consumption recommendations since toxicity to humans depends on tissue concentrations rather than ingestion rates. Similarly, refining our understanding of depuration rates in human hair is important for predicting risks to fish consumers based on previous levels of exposure to Hg.

### The Use of Hg Stable Isotopes to Track MeHg Depuration and Subsequent MeHg Intake

To complement concentration dynamics in the hair of our two subjects, we tested whether stable isotope values could reveal additional insights. Gabonese fish were the sole source of hair mercury during travel, but for epidemiological studies, inorganic Hg also can contaminate hair through exposure to gaseous elemental Hg or Hg-containing personal care products.^9,27^ There are substantial methodological challenges to isolating MeHg prior to Hg isotope analyses,^28,29^ so while we did not need to evaluate iHg contamination because the participants’ history is known, research necessitates that hair samples be tested for speciated MeHg to confirm all hair-Hg originates from dietary exposure to MeHg.

The mercury isotope values of Gabonese fish (Table S2), as well as the differences in baseline isotope ratios between our subjects (Figure 1B–D), created a novel inference space (Figures 2A and S1). Hg isotope values reveal whether exposure is from local fish or long-distance transport of fish purchased at markets.^30–32^ We adopted two applications of Hg isotopes: quantifying MDF from metabolic processes like depuration or detoxification (which are expected to increase δ^202^Hg values^33^) and quantifying tissue assimilation rates following a shift to an isotopically distinct dietary source of MeHg (captured by time series of Δ^199^Hg and Δ^200^Hg; Figure 1C,D).

Shifts in δ^202^Hg values reflect the process of depuration, and initial values can also reveal MeHg sources after accounting for the offset between fish δ^202^Hg values (typically reported to range 1.5‰ to 2.0‰ higher)^16,17^ from metabolic processing. For the frequent fish consumer, we expected the higher initial burden of MeHg to create a mix of two Hg sources following MeHg assimilation in Gabon, but the low initial MeHg of the infrequent fish consumer should have produced Hg isotope values derived exclusively from Gabonese fish. To our surprise, the lowest δ^202^Hg value (−0.59‰) in the infrequent fish consumer’s hair deviated substantially from that predicted (0.36‰) by adding MDF-driven increase in δ^202^Hg (1.75‰) to the Gabonese fish δ^202^Hg value (−1.39 ± 0.27‰) (Figure 1B). This difference (0.94‰) could be due to lower metabolically induced MDF, because we sampled rapidly following fish consumption prior to equilibrium (Figure 2D). Further strengthening the concept that initial decline in δ^202^Hg value is almost entirely attributable to Gabonese fish, the infrequent fish consumer increased approximately 1300 ng g^–1^ HgT DW, or >8-fold, in that 15 day span suggesting only roughly 10% of the elevated concentration is resulting from their original baseline. We estimate it would take an additional 36 ± 3 day equilibria to achieve literature estimated offset values (1.75‰); an unlikely condition in real-world context considering past determination of δ^202^Hg offsets were largely estimated for fish-reliant communities.^16,17,30–32^ We conclude that both recent MeHg intakes and those made in the months preceding need consideration when evaluating δ^202^Hg offsets between fish and fish consumers from depuration-induced MDF.

Using Δ^199^Hg and Δ^200^Hg (Figure 1C,D and Table S2),^19–21^ we confirmed that the pre-expedition hair values align with expectations from prior fish consumption. When the frequent fish consumer first ate fish following the expedition, an aberration in the time series (open symbols) confirms that Hg isotopes are sensitive to even short-lived changes in Hg sources (Figure 1B, day 48). Aided by eq 1 we selected Δ^199^Hg value at the peak influence of Gabonese fish versus the Δ^199^Hg value pre-expedition and the Gabonese fish Δ^199^Hg value to estimate Gabonese fish contributions.

1 $${\Delta^{199}Hg}_{Gabonfish}\times {Fraction}_{Gabon\;fish}+{{\Delta^{199}Hg}_{X}}_{y}\times \left({1-Fraction}_{Gabon\;fish}\right)={\Delta^{199}Hg}_{Hair‐measured\;at\;peak\;influence}$$

*X* and *y* represent the participant and their corresponding Δ^199^Hg value at the height of Gabonese-fish influence, determinable by highest HgT concentration in hair for participant *X*. Fraction_Gabonfish_ is the unknown variable, but we did not rearrange the formula for purposes of clarity. The facial hair of the infrequent fish consumer reflected nearly 95% Gabonese Hg, similar to the 90% estimate made from concentrations alone, whereas the scalp hair of the frequent fish consumer reflected only 63 ± 25% Gabonese Hg, with the remaining attributable to precampaign exposures. These results speak to the novel ability to attribute fractional contributions of dietary MeHg from fish of highly different ecosystems that impart dissimilar Hg isotope compositions in the fish tissue.

## Conclusions

### Considerations When Comparing Fish Tissue δ^202^Hg to the δ^202^Hg in Hair of Fish-Consumers

While limited in participant count (and correspondingly a lacking of a diverse array of vulnerable groups), and lacking the consideration of other MeHg-containing foods (e.g., rice), our study provides new insights into the kinetics of fish-sourced MeHg depuration within the human body. MeHg processing within organisms, including in vivo demethylation^33,34^ and sorption or release processes,^35,36^ exclusively drives MDF. Though we had only two subjects in this study, the parallel effects in the quantity and source of ingested MeHg give us confidence in some key inferences. While literature suggests the MDF attributable to metabolic processes between fish and consumer hair results in an increase of 1.5‰ to 2.0‰ of δ^202^Hg,^16,17,30–32^ our initial δ^202^Hg offset between fish and consumer was 0.9‰, and it took an additional 36 ± 3 days, or a 298 ± 40 ng g^–1^ decline in hair Hg, to reach 1.5‰. In fish-reliant communities, periods of fish abstinence this long are unlikely. We conclude the magnitude of δ^202^Hg offset found in literature results from fractionation during both initial metabolic processing (0.9‰) and the subsequent interplay between depuration of older MeHg and recent MeHg assimilation, yielding higher δ^202^Hg values. The latter likely drives the observed variance in the δ^202^Hg offset between fish consumed and human hair in literature. Alternatively, this variance may relate to poorly characterized Hg speciation in the hair because inorganic Hg can impact Hg isotope results. Ultimately while we may not provide a comprehensive framework for the impact of depuration on Hg isotope values, we provide a pilot concept that future work may consider. Establishment of the impact of hair-Hg depuration on Hg isotopes could yield stronger inference when using human hair in tracking human-MeHg exposure.

Our depuration results suggest that MeHg releases are kinetically regulated yet consistent between individuals that differed sharply in prior exposure, and that there is a limit to MeHg depuration via hair follicles. Our results may not be generalizable but here we observe individuals with similar MeHg exposures, but dissimilar MeHg burden histories respond to a similar level of contamination in hair, rather than the frequent fish consumer being considerably higher. Stable isotope results indicate that MeHg release rates are similar between participants despite contrasting MeHg exposure histories, which is consistent with findings from wildlife species.^25,37^

Again, while limited by the number of participants, these results suggest there exist complexities in human-based biomarker research that may not have been fully considered. For our two participants, instantaneous hair concentrations may not be an accurate reflection of longer term (>1 month) MeHg exposure, especially for the individual with a low starting baseline. Whether this is translatable to community level studies remains to be seen, but future work might consider this context when working within communities whose dietary Hg intake varies seasonally due to access constraints or market flows. Our findings suggest that such inconsistency in dietary exposure may not be reflected by snapshots of hair Hg; people with lower initial Hg burdens may show a higher proportion of recently ingested MeHg, making inferences about longer exposure histories (>1 month) less reliable. Snapshot surveys might be paired with dietary records (timing and intensity of fish consumption) and fish sampling as context before seeking to translate hair MeHg into inferences about recent Hgexposure. Mercury isotopes can provide additional insights into Hg sources to fish, inaccuracies within dietary reporting and unreported exposure to elemental Hg.

## Supplementary Material



## Notes

Five pages containing supporting text, 2 supplemental figures and 2 supplemental tables, references (PDF)
