# Supplementary material for: Quantifying Depuration of Methylmercury from Fish Consumption by Travelers
Source: Environ Health (Wash). 2025 Nov 4;4(2):324–30. doi: 10.1021/envhealth.5c00389 (PMC12930318; doi:10.1021/envhealth.5c00389)
Supplement: Supplementary file 1 [file eh5c00389_si_001.pdf]

Supporting Information

Quantifying depuration of methylmercury from fish consumption by travelers

Ryan F. Lepak[*,a], *Jean Hervé MVE BEH*[b], *Clotaire MOUKEGNI-SIKA*[c], *Jean Noël BIBANG BI NGUEMA*[d], *Sarah E. Janssen*[e], *Jacob M. Ogorek*[e], *Michael T. Tate*[e], *and Peter B. McIntyre*[f]

[a]U.S. Environmental Protection Agency Office of Research and Development, Center for Computational Toxicology and Exposure, Great Lakes Toxicology and Ecology Division, 6201 Congdon Blvd, Duluth, MN 55804, USA

[b]Institut de Recherches Agronomiques et Forestières, CENAREST, Laboratoire d'Hydrobiologie et d'Ichtyologie, BP 2246 Libreville, République Gabonaise

[c]Agence Nationale des Parcs Nationaux, Ministères des Eaux et Forêts, 3641, Libreville, République Gabonaise

[d]Institut de Recherches Agronomiques et Forestières (IRAF), Centre National de la Recherche Scientifique et Technologique (CENAREST), BP 2246, Libreville, Gabon

[e]U.S. Geological Survey, Upper Midwest Water Science Center, USGS Mercury Research Laboratory, 1 Gifford Pinchot Drive, Madison, WI 53705 USA

[f]Department of Natural Resources and the Environment, Cornell University, 226 Mann Drive, Ithaca, NY 14853, USA

[*]corresponding author

[*]Ryan Lepak, U.S. EPA Office of Research and Development, Center for Computational Toxicology and Exposure, Great Lakes Toxicology and Ecology Division, 6201 Congdon Blvd, Duluth, MN 55804, USA – lepak.ryan@epa.gov

Table of contents

Page S3, Figure S1 – $\delta^{202}Hg$, $\Delta^{199}Hg$ and $\Delta^{200}Hg$ values in hair and fish and changes hair isotope values from baseline for the participants.

Page S4, Figure S2 – Shifts in $\delta^{202}Hg$ and $\Delta^{199}Hg/\Delta^{200}Hg$ values in hair from baseline due

Page S5, Table S1 – Quality assurance and control results as well as averaged Gabonese fish results.

Page S5, Table S2 – Hg concentrations and isotope values in the hair of participants.

Supporting text

Determining half-life:

$$C_t = C_0 * e^{-b*t}. \text{ Eqn. 1}$$

Here, C_t and C_0 are the hair concentrations at any time, *t* (in days), following the maximum hair-Hg concentration, and *b* is the constant proportion of MeHg eliminated each day. Once *b* is determined, half-life ($T_{0.5}$) is calculated by:

$$T_{0.5} = \frac{\ln(2)}{b}. \text{ Eqn 2}$$

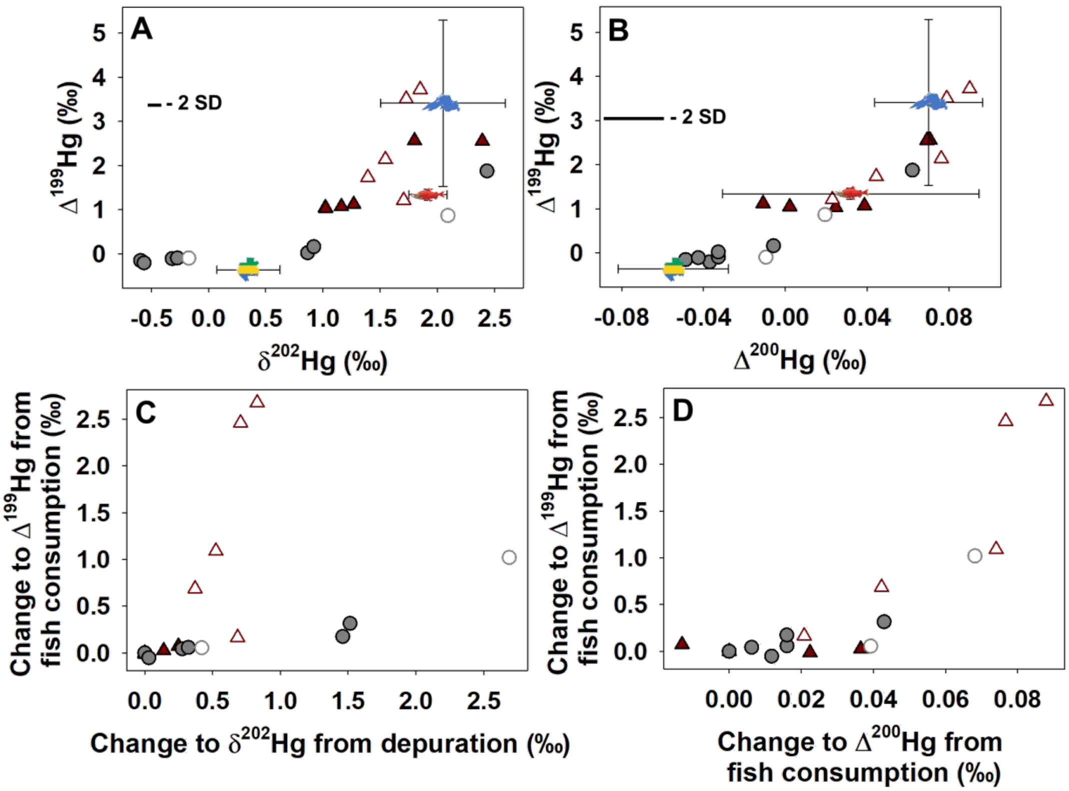

*Figure S1: A (top) - δ^{202}Hg values and Δ^{199}Hg values (‰) in hair and fish. Symbols of Lake Superior,[1, 2] Gabon and an Alaskan sockeye salmon (*Oncorhynchus nerka*)[3] represent fish δ^{202}Hg values and Δ^{199}Hg values from those respective basins. The errors on data symbols represent 1 standard deviation of that data but the standalone error bars represent the 2-standard deviation for this study. Filled triangles and circles represent study-period abstinence from fish consumption for the frequent and infrequent fish consumers respectfully. Empty triangles and circles then represent time periods where fish was consumed infrequently and for the infrequent fish consumer, only once. This formatting is conserved throughout. B (top) – Δ^{200}Hg values and Δ^{199}Hg values (‰) in hair and fish in those media. C (bottom) – Changes from baseline in hair δ^{202}Hg values and Δ^{199}Hg values (‰) resulting from depuration and fish consumption respectfully. D (bottom) - Changes from baseline in hair Δ^{200}Hg values and Δ^{199}Hg values (‰) resulting from fish consumption.*

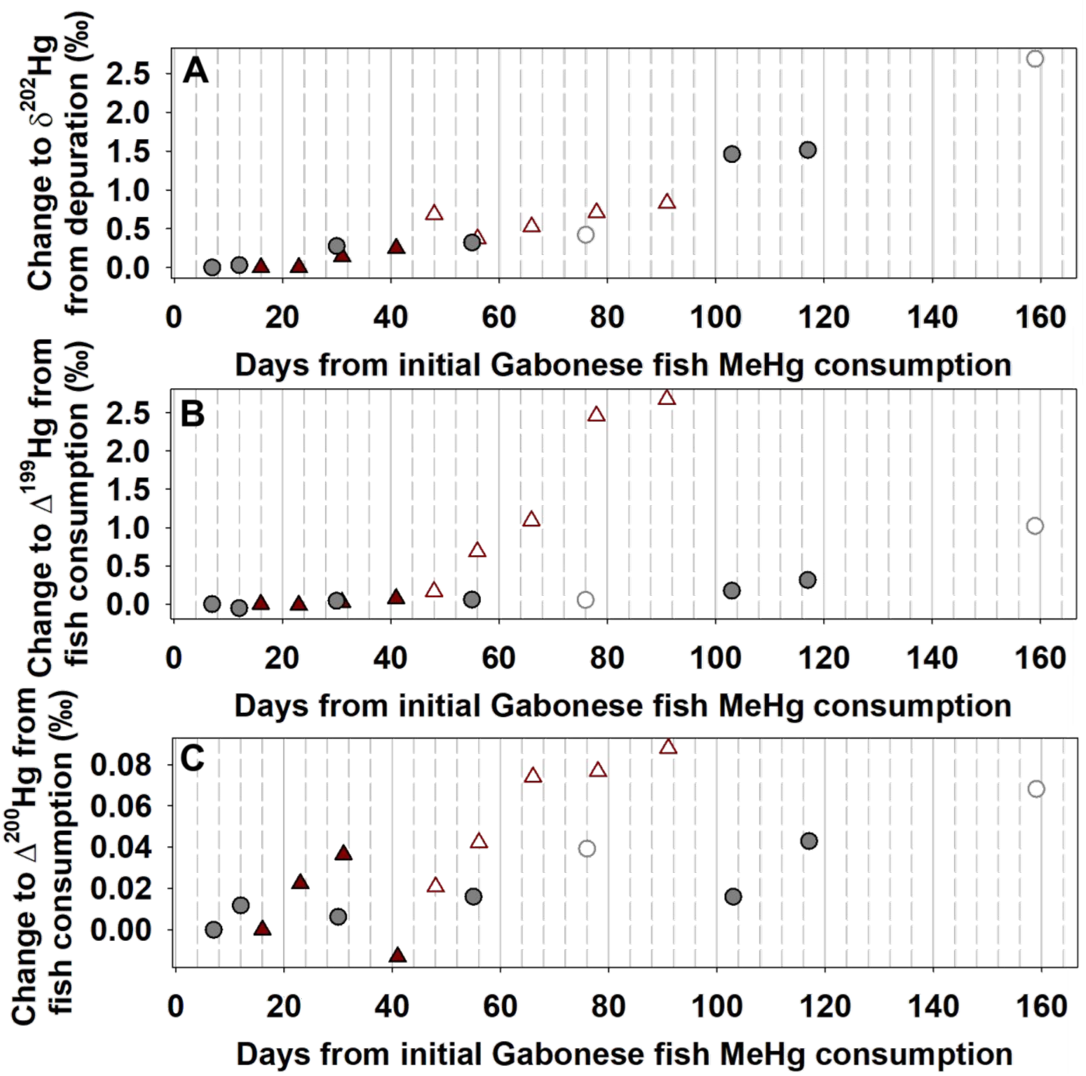

Figure S2: A (top) - Changes from baseline in hair δ^{202}Hg values (‰) from methylmercury (MeHg) depuration. Filled triangles and circles represent study-period abstinence from fish consumption for the frequent and infrequent fish consumers respectfully. Empty triangles and circles then represent time periods where fish was consumed infrequently and for the infrequent fish consumer, only once. This formatting is conserved throughout. B (middle) – Changes from baseline in hair Δ^{199}Hg values (‰) from fish consumption. C (bottom) – Changes from baseline in hair Δ^{200}Hg values (‰) from fish consumption.

Table S1: Quality assurance and control results for two standard reference materials as well as the precision metrics for a sample triplicate. The Gabonese fish reported constitute the averaged results and one standard deviation of interior fish captured and measured during that campaign.

	[HgT] ng g^{-1} DW	% CV	δ^{202}Hg ‰	δ^{202}Hg - 2SD ‰	Δ^{199}Hg ‰	Δ^{199}Hg - 2SD ‰	Δ^{200}Hg ‰	Δ^{200}Hg - 2SD ‰	Δ^{201}Hg ‰	Δ^{201}Hg - 2SD ‰	Δ^{204}Hg ‰	Δ^{204}Hg - 2SD ‰
NIES - 13	4279	2%	2.19	0.12	1.90	0.04	0.06	0.03	1.56	0.01	-0.11	0.04
UM-Almadén	NA	NA	-0.52	0.07	-0.02	0.02	0.01	0.02	-0.04	0.02	-0.01	0.03
MSC680AQ	173	5%	2.44	0.10	1.88	0.04	0.06	0.02	1.46	0.01	-0.18	0.11
Gabonese fish	1058	121%	-1.39	0.27	-0.36	0.12	-0.05	0.03	-0.35	0.10	0.08	0.05
Alaskan Sockeye	175	49%	0.17	0.17	1.34	0.13	0.03	0.06	1.25	0.41	-0.05	0.11
Western Lake Superior fish	304	58%	0.30	0.54	3.41	1.88	0.07	0.03	2.63	1.48	-0.08	0.06

Table S2: Hg concentrations and isotope values in the hair of participants. Date sampled represents the date the hair was cut, not the growth period. The median growth period is reported in days relative to the arrival to Gabon.

USGS Mercury Research Lab ID	Subject	Date sampled	Gabonese arrival Days	[HgT] ng g^{-1} DW	δ^{202}Hg ‰	Δ^{199}Hg ‰	Δ^{200}Hg ‰	Δ^{201}Hg ‰	Δ^{204}Hg ‰	Designation
MSC814AO	Frequent fish consumer	1/1/2019	-5	853	1.80	2.56	0.07	1.96	-0.12	Pre-Gabon
MSC817AO	Frequent fish consumer	1/15/2019	7	773	2.39	2.55	0.07	2.01	-0.15	In Gabon
MSC824AO	Frequent fish consumer	1/24/2019	16	1668	1.02	1.05	0.00	0.81	-0.04	post Gabon
MSC820AO	Frequent fish consumer	1/31/2019	23	1464	1.02	1.03	0.02	0.80	-0.03	post Gabon
MSC819AO	Frequent fish consumer	2/8/2019	31	1407	1.16	1.07	0.04	0.79	-0.07	post Gabon
MSC823AO	Frequent fish consumer	2/18/2019	41	1242	1.27	1.12	-0.01	0.81	-0.01	post Gabon
MSC816AO	Frequent fish consumer	2/25/2019	48	1063	1.71	1.21	0.02	0.95	-0.03	Following consuming fish
MSC822AO	Frequent fish consumer	3/5/2019	56	1470	1.39	1.73	0.04	1.32	-0.10	Following consuming fish
MSC815AO	Frequent fish consumer	3/15/2019	66	1519	1.55	2.14	0.08	1.65	-0.11	Following consuming fish
MSC818AO	Frequent fish consumer	3/27/2019	78	2107	1.73	3.51	0.08	2.72	-0.12	Following consuming fish
MSC821AO	Frequent fish consumer	4/9/2019	91	1650	1.85	3.72	0.09	2.89	-0.12	Following consuming fish
MSC825AO	Infrequent fish consumer	1/1/2019	-10	120	2.38	1.85	0.05	1.46	-0.17	Pre-Gabon - head hair
MSC680AQ-ave	Infrequent fish consumer	1/1/2019	-1	173	2.44	1.88	0.06	1.46	-0.18	Pre-Gabon - beard hair
MSC827AO	Infrequent fish consumer	1/16/2023	7	1466	-0.59	-0.15	-0.05	-0.16	0.04	In Gabon
MSC681AQ	Infrequent fish consumer	1/21/2019	12	1706	-0.56	-0.21	-0.04	-0.22	0.09	post Gabon
MSC670AQ	Infrequent fish consumer	2/3/2019	30	1507	-0.32	-0.11	-0.04	-0.19	0.06	post Gabon
MSC671AQ	Infrequent fish consumer	2/3/2019	55	1002	-0.27	-0.10	-0.03	-0.14	0.01	post Gabon
MSC676AQ	Infrequent fish consumer	3/2/2019	76	1615	-0.17	-0.10	-0.01	-0.17	0.07	post Gabon - consumed a meal of sockeye
MSC672AQ	Infrequent fish consumer	4/15/2019	103	867	0.87	0.02	-0.03	-0.06	-0.05	post Gabon
MSC678AQ	Infrequent fish consumer	4/15/2019	117	755	0.92	0.16	-0.01	0.09	-0.02	post Gabon
MSC675AQ	Infrequent fish consumer	6/6/2019	159	652	2.09	0.87	0.02	0.67	-0.01	post Gabon - consumed sockeye
